# A Case Report of Dengue-Associated Maculopathy With Literature Review

**DOI:** 10.7759/cureus.35937

**Published:** 2023-03-09

**Authors:** Durgavashini Govinda Raju, Norlina Ramli, Rathna Ramayaj

**Affiliations:** 1 Department of Ophthalmology, Faculty of Medicine, Universiti Malaya, University of Malaya Eye Research Centre (UMERC), Kuala Lumpur, MYS; 2 Department of Ophthalmology, Hospital Melaka, Melaka, MYS

**Keywords:** complications of dengue fever, steroid, macula oedema, maculopathy, dengue

## Abstract

We report a case of dengue-associated maculopathy in a healthy adult. A 31-year-old lady with dengue fever presented with both eye sudden, painless blurry vision in the left eye. On examination, the best-corrected visual acuity was 6/18 in her right eye and 2/60 in her left eye. Anterior segment examination was unremarkable. Fundi showed bilateral macula edema with cotton wool spots. Optical coherence tomography (OCT) macula scan was suggestive of bilateral macula edema, with higher severity in the left eye. She was started on intravenous methylprednisolone 500mg once daily for three days. Upon completion of steroids, her right eye visual acuity improved to 6/6 however her left eye vision worsened to 3/60 after six months. Her OCT six months later showed resolved macula edema on both eyes. Ocular manifestation associated with dengue fever is rare but may result in permanent visual impairment. The use of high-dose steroids helps in improving visual acuity.

## Introduction

This article was previously presented as a meeting abstract and e-poster at the 33rd Malaysia-Singapore Joint Ophthalmic Congress 2018 on March 16-18, 2018.

Dengue fever is a hyperendemic mosquito-borne disease, commonly found in tropical and subtropical climate countries [1,2]. Clinical signs of this febrile disease might range from an infection with no symptoms to one that is severe and causes multi-organ dysfunction [2]. It is one of the globally fastest spreading viral illnesses spread by mosquitoes and because of the potentially fatal consequences of this severe infection, it is a serious public health issue [2]. About half of humanity is now in danger due to the exponential increase in dengue prevalence in recent years [2]. Each year, there are reportedly 100-400 million new infections, with tropical Asia and America having the largest densities according to the infection's global distribution [3]. The four serotypes of the dengue virus (DENV 1 to 4) that are linked antigenically are members of the Flavivirus genus and family (Flaviviridae) [1]. It spreads to individuals when an infected female Aedes mosquito bites them, most often the Aedes aegypti mosquito [1]. There is a substantial risk of morbidity and death from dengue fever. The sickness can range in severity from a simple self-limiting febrile illness requiring just outpatient care to severe dengue with plasma leakage, bleeding issues, or multiorgan failure requiring intensive care unit (ICU) therapy and even having fatal outcomes [1]. Ocular manifestation is not common and was previously considered rare. Apart from symptoms of blurred vision, scotoma, metamorphosis, or floaters, dengue ocular illness might also present as sub-conjunctival, vitreous and retinal hemorrhages, posterior uveitis, or optic neuritis [4]. Here, we describe an intriguing case of a patient with severe dengue and hepatitis who also had the uncommon complication of dengue maculopathy.

## Case presentation

A 31-year-old woman with dengue fever was hospitalized after presenting with symptoms of fever, myalgia, arthralgia, vomiting, and abdominal discomfort that had persisted for three days. There were no signs of bleeding. The hematocrit was 31.5% and the lowest platelet count was 47,000 cells/L with a positive dengue serology (dengue IgM and NS1). Otherwise, she had no other health issues. On the third day of her illness, she came to see us with a sudden, painless blurring of vision in both of her eyes, with the left eye being more affected. She denies any eye redness, pain, discharge, and photophobia.

**Figure 1:**
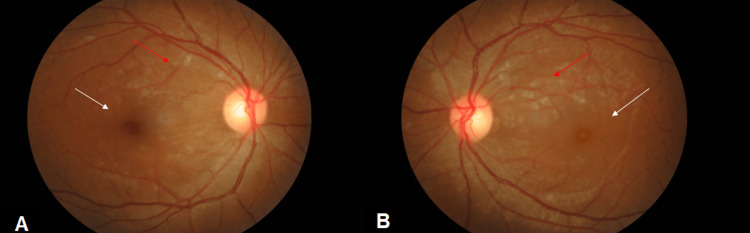
Both eye fundus photo showed bilateral macula edema (white arrow) with cotton wool spots (red arrow) which were worse in the left eye (day 3 of dengue fever) (A) Right eye fundus photo. (B) Left eye fundus photo.

**Figure 2:**
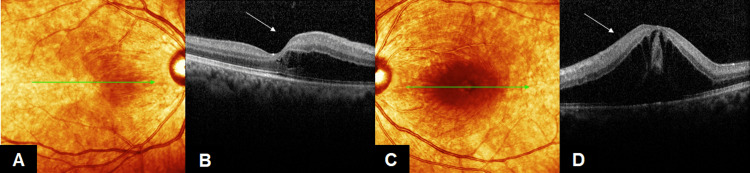
Both eye OCT macula at day 3 of dengue fever (A, B): OCT of RE showed diffuse edema (arrow), (C, D): OCT of LE showed marked subretinal fluid collection (arrow) OCT: Optical Coherence Tomography, RE: Right eye, LE: Left eye

On examination, the best-corrected visual acuity (VA) was 6/18 in her right eye and 2/60 in her left eye. There was no relative afferent pupillary defect. Examination of the anterior segment revealed nothing unusual. A fundus examination revealed cotton wool spots with bilateral macula edema in both eyes (Figures 1A, 1B). Optical coherence tomography (OCT) macula was performed, and the results revealed bilateral macula edema, which was greater in her left eye (Figures 2A-2D).

**Figure 3:**
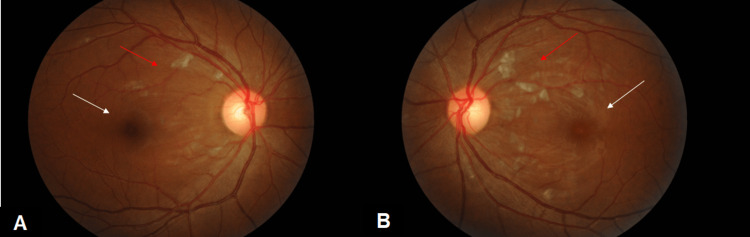
Both eye fundus photo showed cotton wool spots (red arrow) with marked reduction in macula edema (white arrow) at day 4 of dengue fever (A) Right eye fundus photo. (B) Left eye fundus photo.

**Figure 4:**
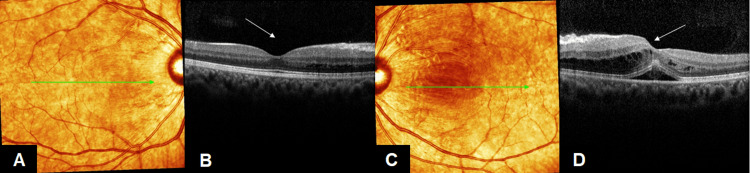
Both eye OCT macula showed a marked reduction in macula edema (arrow) at day 4 of dengue fever (A, B) OCT macula of right eye, (C, D) OCT macula of left eye OCT: Optical Coherence Tomography

The patient was diagnosed as dengue associated maculopathy and was treated with standard of care. She received 500mg of methylprednisolone intravenously once a day for three days. On the second day of treatment, fundus examination and OCT macula showed a marked reduction in macula edema (Figures 3A, 3B, 4A-4D).

**Figure 5:**
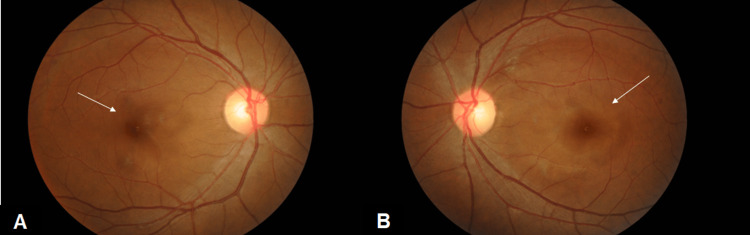
Both eye fundus photo showed resolved macula edema (arrow) after two months of dengue fever (A) Right eye fundus photo. (B) Left eye fundus photo.

**Figure 6:**
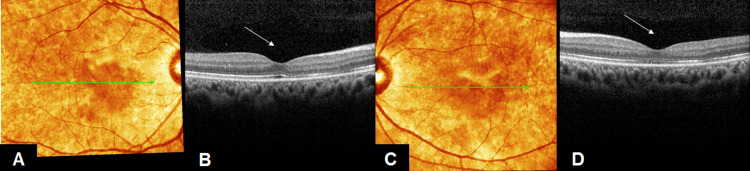
Both eye OCT macula showed resolved macula edema (arrow) after two months of dengue fever. Left eye OCT macula showed a disruption of IS/OS junction. (A, B) OCT macula of right eye, (C, D) OCT macula of left eye OCT: Optical Coherence Tomography, IS/OS: Inner segment/Outer segment

Her fundus and OCT macula two months later showed resolved macula edema on both eyes (Figures 5A, 5B, 6A-6D). Her right eye visual acuity recovered to 6/6 when the steroid treatment was completed, while her left eye vision deteriorated to CF and slightly improved to 3/60 after six months. Patient recovered well systemically with no systemic complications.

## Discussion

Dengue maculopathy manifests as macular edema, hemorrhages, and yellow patches at the macula due to retinal or choroidal vasculopathy [4]. Scotoma, floaters, and impaired vision are among the common symptoms whereas micropsia and metamorphopsia are uncommon [4]. It can manifest as vasculitis, foveolitis, macular hemorrhage, vascular occlusion, or macula edema, among other conditions [4].

Based on the OCT, Teoha et al. further classified the different types of macula edema [5]. Type 1 - diffuse edema, type 2 - cystoid edema, and type 3 - cystic foveolitis are the three patterns that have been identified [5]. Those with cystoid macula edema had worse VA, according to a comparison done between the degree of vision blurring and the type of maculopathy [5]. One or both eyes may be affected by dengue ocular illness, and it can happen as soon as two days or as late as five months after the fever first appears [4].

Nevertheless, it often occurs one day after the lowest point of thrombocytopenia, which is thought to occur seven days following the start of a fever [4]. This is demonstrated by our patient, who began exhibiting symptoms on day 7 while experiencing the disease's lowest platelet count on day 6. The exact pathomechanism of dengue-related ocular complications is unknown, but it may be attributed to an underlying immunological reaction, which may lead to an inflammatory response in retinal tissue and retinal vascular systems [6].

The eye disease's prognosis ranges from full spontaneous recovery to poor eyesight despite treatment [4]. The majority of ocular involvement instances are self-limiting and spontaneously recover without medical intervention [5]. Corticosteroid treatment may be useful for active maculopathy cases with symptoms that have persisted for up to six months [4]. Corticosteroid treatment may aid in the recovery by minimizing structural damage and long-term vision loss brought on by ocular inflammation, as the underlying process is likely immune-driven [7]. In a case series by Fhun et al., one out of three patients who were treated with systemic steroids had visual improvement from CF to 6/9 within three weeks whereas another patient who was treated conservatively had visual improvement from 6/24 to 6/12 within two months [8]. This patient had marked cystoid edema in the left eye and diffuse swelling in the right eye. Intravenous Methylprednisolone improved the macula edema after just one dose. As for the visual acuity, improvement was only seen in the right eye due to the lesser structural damage of its photoreceptors as compared to the left eye. The poorer vision of the left eye is due to the disruption of the IS/OS junction as seen in the OCT macula. The immune complex deposition leading to defective capillary endothelium or blockage at the level of the collecting venule, resulting in ischemia of the choriocapillaris, has been suggested as the most likely underlying pathophysiological mechanism [9]. Further tests such as fundus fluorescein angiography (FFA) or OCT angiography (OCTA) would have been beneficial to detect any presence of macula ischemia or retinal vasculature perfusion as it provides detailed, high-resolution images of the retinal vasculature segmented by layer [10,11]. Unfortunately, FFA and OCTA tests were not available at our center and thus were not done for this patient. 

## Conclusions

In conclusion, dengue fever may cause maculopathy with many other ocular manifestations. Besides OCT and fundus photo, fundus fluorescin angiography, indocyanine green angiography, and visual field tests are all extra tests that can be used in the diagnosis and extent of the severity of this disease. Dengue-associated maculopathy will improve spontaneously with time. Treatment with steroids may help with visual recovery and prevent permanent visual loss. However, structural damage to photoreceptors may halt visual recovery and cause permanent visual loss.
